# A Case of Rapidly Progressive Sensory Neuropathy

**DOI:** 10.7759/cureus.81422

**Published:** 2025-03-29

**Authors:** Chiara Scopice, Jane Louie, Claudia J Chaves, Hiren Patel, Clement Lee

**Affiliations:** 1 Neurology, University of Texas Health Science Center at Houston, Houston, USA; 2 Neurology, Tufts University School of Medicine, Boston, USA; 3 Neurology, Newton-Wellesley Hospital, Newton, USA; 4 Neurosurgery, Newton-Wellesley Hospital, Newton, USA; 5 Internal Medicine/Pediatrics, Tufts University School of Medicine, Boston, USA

**Keywords:** acquired demyelinating disease, acute motor sensory neuropathy, cervical spondylolic myelopathy, degenerative cervical myelopathy, spinal cord disease

## Abstract

A 62-year-old man presented with sensory neuropathy that evolved quickly over the course of weeks, starting with hand involvement and progressing to include the lower extremities and his torso. Nerve conduction studies were consistent with a demyelinating process. Spinal imaging showed cervical spondylotic myelopathy, and urgent surgical nerve decompression was performed.

## Introduction

Cervical spondylotic myelopathy (CSM) is the most common cause of non-traumatic spinal cord disease in adults, with a prevalence of 60.5 per 100,000. It is characterized by progressive compression of the cervical spine due to disc herniation, osteophytes, or spinous ligament ossification [1]. Given its non-specific presentation and overlap with other common conditions, CSM is susceptible to misdiagnosis. This case describes an atypical presentation of CSM with rapidly progressive neuropathy.

## Case presentation

A 62-year-old man with a history of hypothyroidism, hypertension, and B12 deficiency presented with bilateral hand tingling. Symptoms developed over the past few weeks and were associated with loss of hand dexterity while writing and tying shoes. He denied constitutional symptoms, including fevers and chills. There was no temporality to his complaints, and all of his fingers were affected equally.

The patient's medications included valsartan, levothyroxine, and vitamins D and B12. Past surgeries included thyroidectomy and lipoma removal. He denied tobacco or recreational drug use and drank two to three alcoholic drinks weekly. There was no family history of neurologic or autoimmune disease. He lived in Massachusetts and had no occupational exposures.

On examination, both hands appeared grossly normal. Strength was preserved in the flexor and extensor muscles of the hands and forearms bilaterally. There was decreased sensation to touch and vibration of his fingers; pain and temperature testing were not performed. Phalen’s and Tinel’s tests were negative.

The patient’s complete blood count (CBC), comprehensive metabolic panel (CMP), erythrocyte sedimentation rate (ESR), C-reactive protein, and anti-nuclear antibody were all normal. The thyroid-stimulating hormone level was 0.20 uIU/mL (reference range, 0.35-5.50), and his levothyroxine dose was adjusted. A hemoglobin A1C level was 5.4% (reference range, 4.3-5.6%) and B12 level was within normal limits (877 pg/mL; reference range, 211-911 pg/mL), with normal methylmalonic acid levels. No abnormalities were detected on serum and urine protein electrophoresis. These laboratory studies ruled out diabetes mellitus, B12 deficiency, systemic lupus erythematosus, hypothyroidism, and paraproteinemia as causes of his neuropathy. At this point, carpal tunnel syndrome was thought to potentially explain his symptoms.

One week later, nerve conduction studies (NCS) showed reduced sensory responses and focal motor conduction slowing in the left ulnar nerve. There was no conduction block, and compound muscle action potentials (CMAPs) were normal in amplitude and morphology. Motor and sensory studies of the right ulnar nerve and bilateral median nerves were normal. Electromyography (EMG) showed large motor unit action potentials (MUAPs), chronic denervation-reinnervation changes in the left first dorsal interosseous and deltoid muscles, and decreased recruitment of large motor units. These studies did not support a diagnosis of carpal tunnel syndrome, and several autoimmune neuropathies (e.g., sarcoidosis, paraneoplastic syndromes, chronic inflammatory demyelinating polyneuropathy, and multiple sclerosis) and compressive neuropathies remained on the differential.

Two weeks after initial presentation, the patient reported new decreased sensation in both of his feet that ascended to his lower abdomen. He denied fecal or urinary incontinence. On examination, there was decreased sensation to touch and vibration of his fingers up to the shoulders and of his feet up to the lower abdomen. Pain and temperature sensation were not affected. Deep tendon reflexes were 3+ throughout except at the ankles. There was mild left pronator drift. His gait was broad-based, and Romberg testing was positive. Given his rapidly progressive symptoms, he was referred to the emergency room.

**Figure 1:**
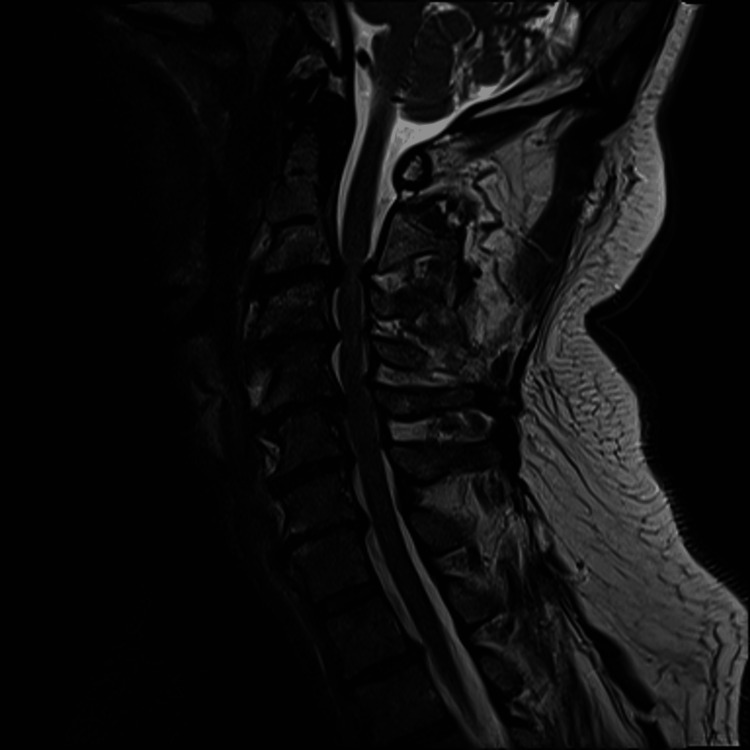
Sagittal T2-weighted MRI of the cervical spine.

**Figure 2:**
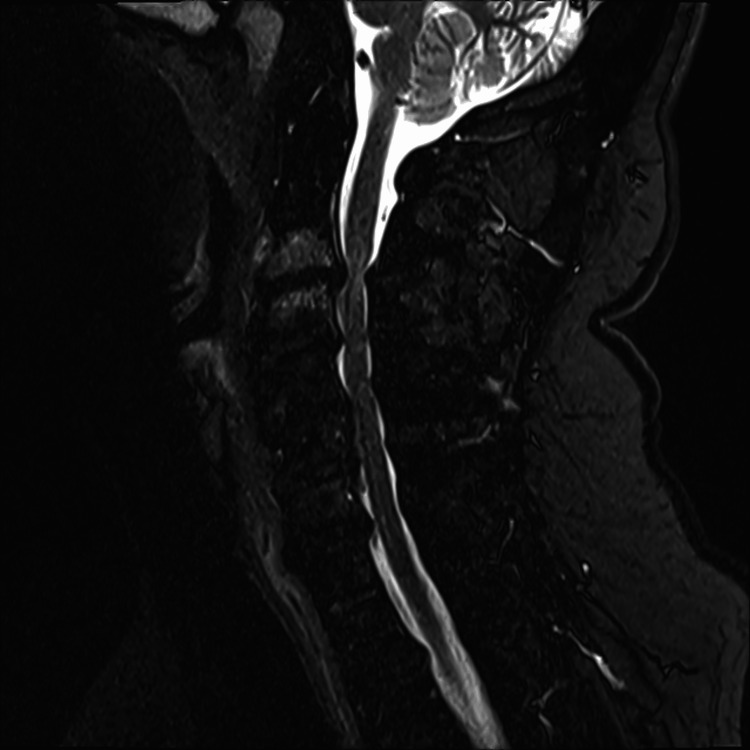
Sagittal short tau inversion recovery (STIR) MRI of the cervical spine.

A non-contrasted MRI of the cervical spine was obtained (Figures 1, 2). The MRI showed severe cervical spinal canal stenosis with cord compression at C3-C4 and C5-C6, with signal change at C3-C4. There was bilateral facet arthropathy of C3-T1, resulting in various degrees of foraminal stenosis.

**Figure 3:**
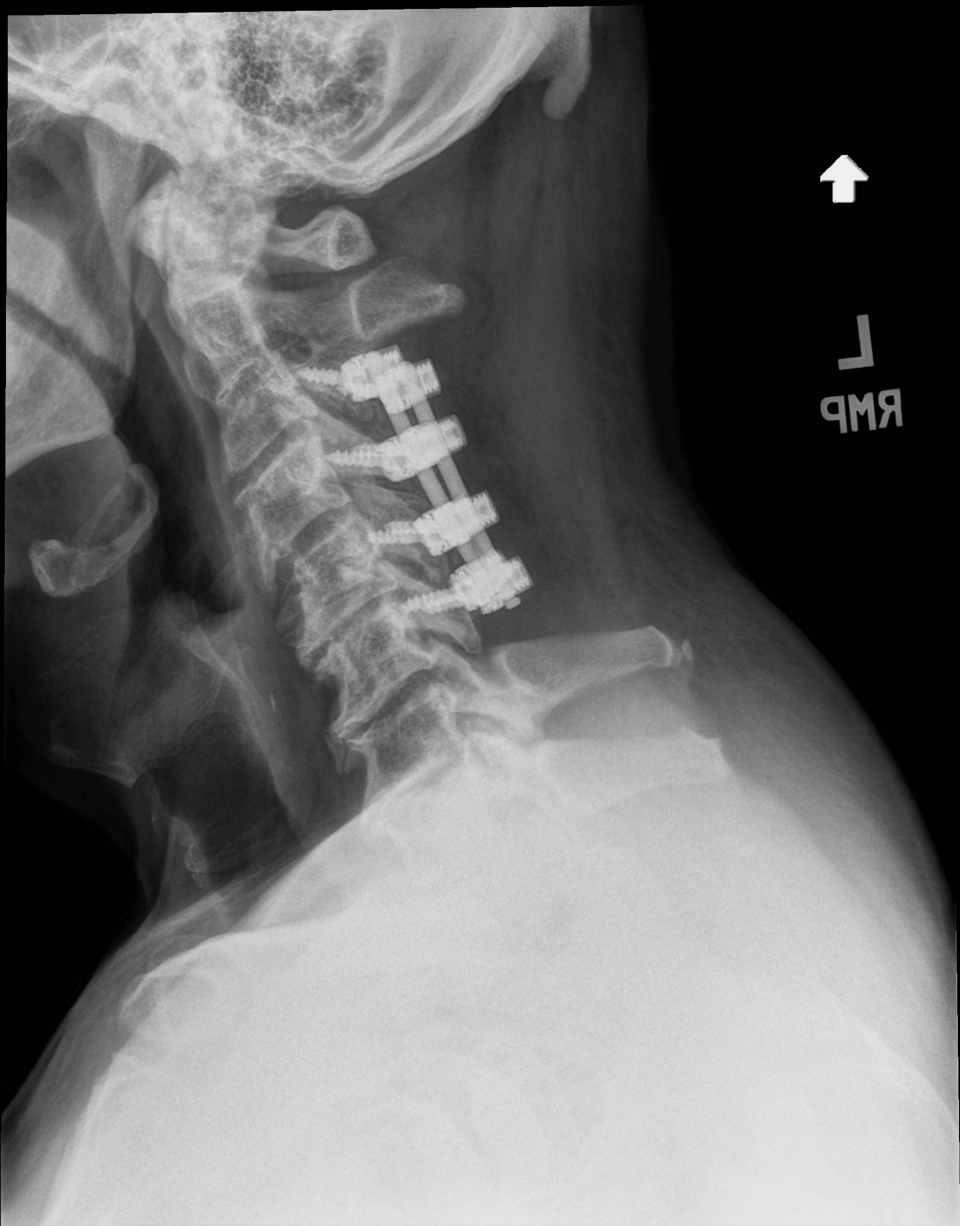
Postoperative radiography showing posterior spinal decompression and fusion spanning C3-C6.

The patient underwent urgent C3-C6 laminectomy, foraminotomy, and arthrodesis (Figure 3). Following surgery and physical therapy, sensation was regained in the upper and lower extremities, with improvement in range of motion and mild residual loss of dexterity. One year after surgery, he has not experienced any recurrence of his initial symptoms, and long-term follow-up is planned.

## Discussion

We describe the case of a 62-year-old man who presented with rapidly progressive sensory and motor neuropathy, with demyelinating features on nerve conduction studies, who was ultimately found to have cervical spondylotic myelopathy (CSM). This case was notable for the findings of demyelination on NCS, which may have delayed the diagnosis of CSM. CSM is usually associated with a normal NCS [2]. In rare cases where CSM is associated with abnormal NCS, signs of radiculopathy are the most common finding, in contrast with the findings in our patient [3]. Expert consensus guidelines have noted that electrophysiological tests, including NCS, “are useful in differential diagnosis of CSM,” but do not strongly recommend for or against their routine use [4]. Demyelination has been described in patients with CSM, which may explain the findings and symptoms in our patient [5]. Microstructural changes in myelin have been documented in spinal compression, and this may translate to compromised transmission of fine touch and vibration via myelinated Aβ nerve fibers.

A second unique feature of this case was the rapid progression of CSM, a disease that usually evolves slowly. Patients with rapid disease, defined as difficulty standing or walking within four weeks of symptom onset, appear to have better neurological recovery after decompression compared to those with slower progression [6]. Prior descriptions of rapid progression in CSM typically involve motor deficits, and pure sensory involvement, as seen in our patient, has not been specifically described [6-7]. However, many of the studies quantified progression using the modified Japanese Orthopedic Association (mJOA) scale, which includes sensory symptoms [6-7]. Involvement of C3-C4 has been associated with rapid progression, as seen in our patient [7]. Other reported risk factors include congenital spinal stenosis, diabetes mellitus, hypertension, and prior cerebrovascular events [6, 8]. Interestingly, in one analysis, facet joint irregularity was identified as the most important risk factor for rapid progression, and our patient’s widespread facet arthropathy may have contributed to his unique presentation [8].

CSM can be managed surgically or conservatively. Conservative management is preferred in asymptomatic patients and includes cervical immobilization, pain medications, and lifestyle modifications to avoid whiplash or falls [9]. If symptoms of radiculopathy are present, the risk of myelopathy should be discussed, as early management is associated with better prognosis.

Surgical intervention for CSM involves decompressing and/or stabilizing the spinal cord and is recommended for patients with moderate to severe disease, based on clinical guidelines [10]. In our patient, who had cord signal abnormality and severe, rapidly progressive symptoms, surgical intervention was indicated. Surgical decompression shortens the duration of symptoms in most patients [10]. Common surgical approaches target either the anterior or posterior vertebrae and include cervical discectomy and laminectomy, respectively. The specific approach depends on the location of myelopathic changes on MRI, anatomical variations, and surgeon expertise. Overall, the prognosis of patients undergoing surgery for CSM is excellent. In long-term follow-up studies, 90% of patients report stability or improvement in symptoms [11].

## Conclusions

Cervical spondylotic myelopathy is a common degenerative disease that usually presents with chronic motor or sensory myeloneuropathy. In rare cases, it may present acutely with rapidly progressive symptoms, mimicking other neurologic diseases. Prompt imaging and treatment are warranted to improve surgical outcomes. Specifically, early spinal imaging is important to consider when nerve conduction studies or other data do not elucidate a clear neurologic cause of disease.
